# Prevalence of Iron Deficiency and Iron Deficiency Anemia Among Adolescent Girls at a Tertiary Care Centre in Bihar: A Cross-Sectional Study

**DOI:** 10.7759/cureus.87078

**Published:** 2025-06-30

**Authors:** Haripriya H, Neeraj Agrawal, Pragya Kumar, Alok Ranjan, Shamshad Ahmad, Sushil Kumar, Neha Chaudary, Sarita Kumari, Venkatesh Karthikeyan

**Affiliations:** 1 Community and Family Medicine, All India Institute of Medical Sciences, Patna, Patna, IND; 2 Community and Family Medicine, All India Institute of Medical Sciences, Bibinagar, Bibinagar, IND; 3 Biochemistry, All India Institute of Medical Sciences, Patna, Patna, IND; 4 Community Medicine, ESIC (Employees' State Insurance Corporation) Medical College and Hospital, Bihta, Patna, IND

**Keywords:** adolescent, anemia, anemia mukt bharat, ferritin, hemoglobin, iron-deficiency, iron deficiency anemia (ida)

## Abstract

Background

Iron deficiency (ID) and iron deficiency anemia (IDA) are significant public health concerns, particularly in low- and middle-income countries. Adolescents, especially girls, are at a heightened risk due to increased iron requirements during growth and menstruation.

Objective

This study aimed to estimate the hospital prevalence of ID and IDA among adolescent girls attending the Outpatient Department (OPD) at a tertiary care centre in Bihar, India.

Methods

A hospital-based cross-sectional study was conducted at the All India Institute of Medical Sciences (AIIMS), Patna, India, from June 2019 to October 2021. A total of 169 adolescent girls aged 10 to 19 years were recruited using a non-probability convenient sampling method. Participants with normal C-reactive protein (CRP) levels were included to ensure accurate assessment of iron status, without the influence of inflammation. Serum ferritin, hemoglobin, serum iron, total iron-binding capacity (TIBC), and percentage saturation were measured. The primary outcomes were the prevalence of ID (serum ferritin < 15 µg/L) and IDA (serum ferritin < 15 µg/L and hemoglobin < 12 g/dL).

Results

The hospital prevalence of ID among the study participants was 17.8% (95% CI: 11.8%-23.5%), while the hospital prevalence of IDA was 15.4% (95% CI: 10.1%-21.7%). The overall hospital prevalence of anemia was 47.9% (95% CI: 40.2%-53.8%). A higher hospital prevalence of ID and IDA was observed in older adolescents (aged 17-19 years) compared to younger ones (aged 10-16 years), though the association was not significant (p = 0.056).

Conclusions

The findings of this study revealed that almost half of the adolescent girl population had a high hospital prevalence of anemia, with the majority of these cases classified as mild to moderate in intensity, whereas the hospital prevalence of absolute ID and IDA was low. Early screening using serum ferritin and appropriate nutritional strategies is recommended to address this public health issue.

## Introduction

In the fight against malnutrition, iron deficiency (ID) and iron deficiency anemia (IDA) stand as major threats, particularly in India, where adolescent girls are disproportionately affected, resulting in adverse health outcomes. ID represents a silent epidemic, affecting a staggering one-third of the global population. According to the World Health Organization (WHO), 53% of non-pregnant women aged 15-49 years in India suffered from anemia in 2019, and the National Family Health Survey-5 (NFHS-5, 2019-2021) reported the prevalence of anemia among adolescent girls aged 15-19 years at 59.1%, with an even higher prevalence of 65.7% in Bihar [1-3].

Anemia is a condition characterized by a lack of sufficient red blood cells, which is widely recognized and measured by estimating the hemoglobin level in blood. Nutritional deficiencies, such as ID; deficiencies in folate, vitamin B12, and vitamin A; along with hemoglobinopathies and infectious illnesses like malaria, tuberculosis, human immunodeficiency virus (HIV), and parasitic infections, are among the major causes of anemia [4,5]. The most frequent micronutrient shortage in the world is iron insufficiency, which ultimately leads to IDA [6].

Adolescents are particularly vulnerable to ID due to increased iron requirements during growth spurts and, in the case of girls, due to monthly menstrual blood loss. ID affects around 25% of adolescents globally, with significant cognitive and physical impacts, including impaired attention and memory, which can adversely affect academic performance and overall quality of life [6]. Additionally, ID can lead to decreased physical work capacity and endurance - particularly debilitating for adolescents engaged in physical activities and sports [7].

The Comprehensive National Nutrition Survey (CNNS) 2016-2018 indicated that ID prevalence was significantly higher in adolescent girls (30.4%) compared to boys (11%-15%). The prevalence was particularly high in urban areas and among those from wealthier families, potentially due to dietary factors and reduced iron absorption [8].

ID progresses through distinct stages, starting with the depletion of iron stores, leading to iron-deficient erythropoiesis, and ultimately resulting in IDA [9]. This progression underscores the need to detect and address ID before it advances to IDA, as many adolescents remain in a state of ID, suffering from its detrimental effects on their health and development.

At the community level, screening for IDA is typically conducted by measuring the hemoglobin level in the blood. However, this method does not provide an accurate depiction of anemia caused by ID. To accurately diagnose IDA, an iron profile test must be performed. Currently, there is a significant gap in affordable and accessible diagnostic tests for the screening and early detection of ID in India. Serum ferritin is the most sensitive biomarker of ID and is the gold standard for diagnosing ID, providing a clear indication of depleted iron stores, which helps in identifying adolescents at risk before they progress to anemia [8]. Despite this advantage, it is rarely used in routine screenings [10].

Although numerous public health initiatives have been undertaken, progress in reducing the prevalence of anemia has been limited. Relying solely on hemoglobin levels to screen for anemia misses a significant portion of the population who have ID without anemia. Other important biomarkers to detect IDA include transferrin saturation, serum iron, total iron-binding capacity (TIBC), and soluble transferrin receptor (sTfR) levels. Reticulocyte hemoglobin content (CHr) is a more accurate early marker of iron-deficient erythropoiesis, making it a better indicator of ID compared to hemoglobin alone [11]. The zinc protoporphyrin/heme (ZPP/H) ratio is another sensitive and specific test for early iron depletion before the onset of anemia. It is simple to perform and cost-effective, making it suitable for community screening [12].

Additionally, dried blood spot assays, which involve collecting capillary blood on filter paper for later analysis of various iron markers, are practical for use in field settings and remote areas [12]. Innovations like the ironPhone provide portable diagnostics for quantifying serum ferritin concentrations from a drop of finger-prick blood, enabling rapid and accurate assessment of iron status at the point of care [13].

These methods enhance the feasibility of screening for ID in community settings, facilitating early detection and intervention to prevent the adverse effects of ID on adolescent health and development. Furthermore, identifying the true prevalence of ID helps in designing targeted nutritional programs and public health strategies, ensuring that interventions are appropriately focused on those who need them the most, thereby improving their effectiveness.

The current hospital-based study aims to estimate the hospital prevalence of ID and IDA among adolescent girls attending the tertiary care centre in Patna, Bihar.

## Materials and methods

Study design and setting

This hospital-based cross-sectional study was conducted at the All India Institute of Medical Sciences (AIIMS), Patna, a prominent tertiary care centre located in the capital city of Bihar. The institute has a total bed capacity of 960, catering to all types of emergency and high-risk cases. The study took place over two years and four months, from June 9, 2019, to October 29, 2021.

Participants

Adolescent girls aged 10 to 19 years, attending the Outpatient Department (OPD) of Paediatric Medicine and Obstetrics & Gynaecology (OB&G) at AIIMS Patna, with normal levels of C-reactive proteins (CRPs) in their blood and who expressed willingness to participate, were enrolled in the study. Elevated CRP levels were considered indicative of chronic conditions such as bacterial infections (e.g., sepsis) and fungal infections, ensuring the exclusion of acute etiologies of anemia. Exclusion criteria included a recent history of blood transfusion or blood donation within the past three months; major surgical procedures or significant blood loss due to recent accidents within the past six months; and a history of chronic diseases such as hypertension, diabetes mellitus, tuberculosis, cardiac diseases, and chronic kidney disease.

Operational definitions

ID was operationally defined as a serum ferritin value <15 µg/L, indicating reduced iron stores in the pre-latent stage, before the complete manifestation of IDA. IDA was defined by a serum ferritin value <15 µg/L, along with a hemoglobin value <12 g/dL [14,15].

Sample size and sampling technique

The estimated sample size of this study was 169, which was calculated using the formula: $n = \frac{Z^2 \cdot p \cdot (1 - p)}{d^2}$, where Z = reliability coefficient (value = 1.96), p = prevalence, and d = absolute error. The prevalence (p) was taken as 22%, based on the prevalence of ID in adolescent girls aged 10-19 years from the CNNS (2016-2018) [16]. Keeping a 30% relative margin of error in the measured prevalence and a 95% confidence interval, the minimum sample size was calculated to be 152. Assuming a 10% nonresponse rate and abnormal CRP estimates, the final sample size was determined to be 169. The study participants were recruited using a non-probability convenient sampling technique, after obtaining ethical clearance from the Institutional Ethics Committee (IEC) of AIIMS, Patna (approval no. AIIMS/Pat/IEC/PGTh/July19/11).

Study procedure

A semi-structured questionnaire was created and validated through pilot testing prior to data collection (see Appendix). The questionnaire was developed in consultation with four subject experts, and modifications were made following the pilot testing. The study was formally presented to the Heads of the Paediatric Medicine and OB&G Departments. Information was disseminated to other consultants, both senior and junior doctors, as well as department attenders, who were the initial point of contact for patients attending the OPD.

Patient slips containing details such as a unique hospital registration number (CR number), age, and gender - written in English and Hindi - were distributed to all adolescent girls visiting these OPDs, with the help of the staff posted in the respective departments, to maintain confidentiality. The parents/caregivers of adolescent girls meeting the inclusion and exclusion criteria were informed about the study's purpose by the consulting doctor. Written consent was obtained from participants above 18 years of age, and written assent was obtained from the guardians of those below 18 years of age.

Participants were instructed to undergo blood sample collection to investigate their iron profile. Following aseptic procedures, 6 mL of venous blood was collected from all enrolled adolescent girls free of cost. The blood samples were analyzed for serum ferritin, serum iron, serum TIBC, hemoglobin, and CRP in the Central Laboratory of the Institute using high-quality, standard techniques. The following day, the reports were gathered and disseminated to the participants. Participants with low serum ferritin levels and below-normal values of hemoglobin and other iron profiles were recommended to initiate oral iron supplementation as per the guidelines. Those with solely abnormal iron profiles were referred to the General Medicine Department for additional consultation and interventions, while those with normal hemoglobin and iron profiles were advised to maintain an iron-rich diet and continue taking oral iron supplements.

Laboratory analysis

The blood samples were processed on the same day at the Central Laboratory using standard estimation techniques. CRP levels in serum were estimated qualitatively using Randox (RX) series equipment (Randox Laboratories, Crumlin, Northern Ireland). The study excluded participants with elevated CRP levels. The normal range of CRP is 0-5 mg/L, as per the institutional guidelines followed by the Department of Biochemistry. Serum ferritin values were estimated using calibrated Beckman Coulter Chemistry Analyzers AU680 and AU2700/5400 (Beckman Coulter, Inc., Brea, CA, USA). Serum iron and TIBC were estimated using the RX series analyzer (RX Suzuka, Cat. No. SI 8049) and the calorimetric method with RX series instruments (Cat. No. Tl 4064).

Statistical analysis

Data were entered into Microsoft Excel (Microsoft® Corp., Redmond, WA, USA), cleaned, and categorized according to standard operational definitions. Analysis was performed using IBM SPSS Statistics for Windows, Version 22 (Released 2013; IBM Corp., Armonk, NY, USA). The independent variables of the study were the sociodemographic profile of the participants and the laboratory investigation measures, whereas the prevalence of anemia, ID, and IDA were the dependent variables. Descriptive statistics were used for categorical variables like sociodemographic details of the participants and prevalence of anemia, ID, and IDA, which are represented as frequency (percentage), while continuous variables like hemoglobin levels and iron profile are represented as mean ± standard deviation (SD) or median and interquartile range (IQR), considering the normality of the data. Pearson’s chi-square test or Fisher’s Exact test determined associations between categorical variables, with significance set at p < 0.05. The strength of association was measured using Phi and Cramer’s V values (0.1 as weak, 0.3 as moderate, and 0.5 and greater as strong). Parametric (Student’s t-test) or non-parametric tests (Mann-Whitney U test) compared means or medians of continuous variables, considering a p-value < 0.05 as statistically significant. Levene’s test for equality of variances assessed variance equality between groups.

## Results

A total of 169 adolescent girls who visited the OPDs of Paediatrics and OB&G, fulfilling the inclusion and exclusion criteria, were enrolled in the current study. The baseline socio-demographic details of the study participants are shown in Table 1.

**Table 1:** Sociodemographic profile of the study participants (N = 169) *Classification of socio-economic scale was done using Modified B.G. Prasad classification [17].

Characteristics	Frequency (n)	Percentage (95% confidence interval)
Residence		
Rural	90	53.3 (46.2 - 61.4)
Urban	79	46.7 (38.6 - 53.8)
Religion		
Hindu	115	68.0 (60.9 - 75)
Muslim	54	32.0 (25 - 39.1)
Caste		
General	69	40.8 (32.5 - 49.1)
Other backward caste	76	45.0 (37.3 - 54)
Scheduled caste	24	14.2 (8.9 - 18.9)
Location of school		
Rural	73	43.2 (35.7 - 51.6)
Urban	96	56.8 (48.4 - 64.3)
Educational status of the father		
Illiterate	34	20.1 (13.3 - 27.1)
Primary	2	1.2 (0.0 - 3.0)
Middle	15	8.9 (4.7 - 14)
Secondary	41	24.3 (17.8 - 31.4)
Higher Secondary	20	11.8 (7.1 - 16.6)
Graduate	42	24.9 (18.2 - 31.4)
Postgraduate	15	8.9 (5.3 - 13)
Educational status of the mother		
Illiterate	48	28.4 (22.5 - 36.1)
Primary	16	9.5 (4.3 - 14.0)
Middle	33	19.5 (13 - 23.5)
Secondary	45	26.6 (20.1 - 33)
Higher Secondary	12	7.1 (3.7 - 11.7)
Graduate	9	5.3 (2.4 - 9.3)
Postgraduate	6	3.6 (1.2 - 6.9)
Occupation of the father		
Unemployed	41	24.3 (17.2 - 30.2)
Unskilled/Laborer	17	10.1 (5.9 - 15.4)
Semi-skilled	56	33.1 (26.6 - 39.6)
Skilled	22	13.0 (8.9 - 18.2)
Professional	33	19.5 (13 - 26.9)
Occupation of the mother		
Unemployed/Homemaker	143	84.6 (78.9 - 89.9)
Unskilled/Laborer	19	11.2 (6.5 - 16)
Skilled	0	0
Professional	0	0
Family type		
Nuclear	68	40.2 (32.7 - 46.6)
Joint	101	59.8 (53.4 - 67.3)
Socio-economic status*		
Upper class	24	14.2 (9.5 - 19.4)
Upper middle class	34	20.1 (14.4 - 25.9)
Middle class	27	16.0 (11.2 - 21.7)
Lower middle class	56	33.1 (25.6 - 40.8)
Lower class	28	16.6 (11.4 - 22.5)
Marital status		
Married	66	39.1 (31.1 - 47.3)
Unmarried	103	60.9 (52.7 - 68.9)

According to Table 1, among 169 adolescent girls, 53.3% resided in rural areas, and 68.0% followed Hinduism. Most (45.0%) were from other backward castes, and 56.8% attended schools in urban areas. Fathers' education varied, with 24.9% being graduates, 24.3% completing secondary education, and 20.1% illiterate. Mothers' education ranged from illiteracy (28.4%) to secondary education (26.6%). Fathers were primarily semi-skilled workers (33.1%), while 84.6% of mothers were homemakers. Most girls (59.8%) lived in joint families, and 33.1% belonged to the lower-middle socio-economic class. Notably, 39.1% of school-going girls were married by age 19.

Table 2 illustrates the association between age categories and the prevalence of ID and IDA among adolescent girls. The adolescent girls’ age groups were categorized as 10-16 years and 17-19 years, based on the distribution of study participants across the 10-19 years age range. Among girls aged 10-16 years, 16 (14.3%) had ID, while 96 (85.7%) did not. In contrast, among girls aged 17-19 years, 14 (24.6%) had ID, while 43 (75.4%) did not. The chi-square value for this association is 2.73, with a p-value of 0.098, indicating no statistically significant association between age and ID (p-value > 0.05).

**Table 2:** Association of age categories with iron deficiency and iron deficiency anemia in adolescent girls (N = 169)

Age categories	10-16 years, n (%)	17-19 years, n (%)	Chi-square value	p-value
Iron deficiency				
Present	16 (14.3%)	14 (24.6%)	2.73	0.098
Absent	96 (85.7%)	43 (75.4%)		
Iron deficiency anemia				
Present	13 (11.6%)	13 (22.8%)	3.64	0.056
Absent	99 (88.4%)	44 (77.2%)		

Regarding IDA, 13 (11.6%) girls aged 10-16 years had IDA, while 99 (88.4%) did not. Among girls aged 17-19 years, 13 (22.8%) had IDA, while 44 (77.2%) did not. The chi-square value for this association is 3.64, with a p-value of 0.056, suggesting no significant association between age and IDA (p-value close to 0.05). These results indicate a higher prevalence of both ID and IDA among older adolescent girls (17-19 years) compared to younger ones (10-16 years). While the association between age and ID is not statistically significant, similarly, there was also no significant association between age and IDA.

The normality of the laboratory values was assessed by visually examining the Q-Q plot. All parameters, with the exception of TIBC, exhibited a normal distribution. The descriptive statistics for various laboratory parameters among the adolescent girls displayed the mean (±SD) values for hemoglobin, serum iron, serum ferritin, percentage saturation of iron, and unsaturated iron binding capacity (UIBC) as 11.38 (±1.69) g/dL, 58 (±27.4) μg/dL, 33 (±25.38) ng/dL, 15.2% (±9.76), and 15% (±9.86), respectively. The median value (IQR) of total iron binding capacity (TIBC) was 380 (66.4) μg/dL. Table 3 presents a comparison of the average and median values of the laboratory parameters among different age groups of the study participants (N = 169). The median (IQR) of TIBC was higher in the 17-19 age group compared to the 10-16 age group, with a value of 387 (±80). The Mann-Whitney U test, a non-parametric test, was used to compare the median and IQR of TIBC across different age categories. The results did not indicate any statistically significant difference in the median values. However, when analyzing the mean difference of serum iron across the age categories, an independent t-test revealed a statistically significant difference, with a p-value of 0.016 and a t-value of 2.42. Levene's test was used to assess the equality of variances for serum iron levels across different age categories. The test yielded an F-value of 0.045 and a p-value of 0.833.

**Table 3:** Comparison of mean/median of laboratory parameters across age categories of adolescent girls (N = 169) *Statistically significant with a p-value < 0.05.

Age categories (N = 169)	Serum iron: mean (± standard deviation)	Serum ferritin: mean (± standard deviation)	Percentage saturation: mean (± standard deviation)	Total iron binding capacity: median (± interquartile range)	Unsaturated iron binding capacity: mean (± standard deviation)	Hemoglobin: mean (± standard deviation)
10-16 years	61.62 (±26.17)	32.72 (±21.59)	14.60 (±9.64)	379.5 (±64)	14.39 (±9.70)	11.68 (±1.44)
17-19 years	50.96 (±28.42)	33.60 (±31.74)	16.40 (±9.96)	387 (±80)	16.13 (±10.04)	10.82 (±2.00)
T-value/Mann-Whitney	2.42	0.187	1.13	3240	1.808	2.80
p-value	0.016*	0.852	0.258	0.873	0.278	0.006*

The mean values of hemoglobin across the age categories were compared using an independent t-test, which is a parametric test. Levene's test for equality of variances yielded an F-value of 14.83, with a p-value of 0.001, indicating that the assumption of equal variances was violated. The independent t-test yielded a value of 2.80, indicating a statistically significant difference in the mean values of hemoglobin across the age categories, supported by a p-value of 0.006. None of the other laboratory measurements, such as serum ferritin, percentage saturation, TIBC, and UIBC, exhibited any statistically significant differences, as indicated by p-values greater than 0.05.

Among adolescent girls (N = 169), the estimated prevalence of ID was 30 (17.8%), the estimated prevalence of IDA was 26 (15.4%), and the estimated prevalence of anemia was 88 (47.9%), as shown in Table 4.

**Table 4:** Distribution of adolescent girls based on the prevalence of iron deficiency, iron deficiency anemia, and anemia (N = 169)

Variables (N = 169)	Frequency (n)	Percentage (95% confidence interval)
Iron deficiency		
Present	30	17.8 (11.8 - 23.5)
Absent	139	82.2 (76.5 - 88.2)
Iron deficiency anemia		
Present	26	15.4 (10.1 - 21.7)
Absent	143	84.6 (78.3 - 89.9)
Anemia		
Present	88	47.9 (40.2 - 53.8)
Absent	81	52.1 (46.2 - 59.8)

Table 5 illustrates that the proportion of ID prevalence was higher among adolescent girls with moderate anemia (14, or 56%) compared to those with mild anemia (10, or 16.4%). A chi-square test of association was conducted to assess the relationship between ID status and the severity of anemia. The results revealed a statistically significant association, with a Pearson chi-square value of χ² (df = 2) = 16.920 and a p-value of 0.0001. The phi coefficient, φ = 0.5, demonstrated a strong association between the severity of anemia and ID status.

**Table 5:** Association of iron deficiency status with anemia and severity of anemia among adolescent girls (N = 169) *Statistically significant with a p-value < 0.05.

Anemia (N = 169)	Iron deficiency status			
	Present	Absent	Chi-square value	p-value
Present	62 (70.5%)	126 (29.5%)	17.492	0.001*
Absent	77 (95.1%)	4 (4.9%)		
Severity of anemia (N = 88)	Iron deficiency status			
	Present	Absent	Fisher's Exact value	p-value
Mild	10 (16.4%)	51 (83.6%)	-	0.0001*
Moderate	14 (56%)	11 (44%)		
Severe	0	2 (100%)		

## Discussion

Given the high prevalence of nutritional anemia in low- and middle-income countries like India, where ID has been identified as the main cause, it is crucial to accurately evaluate the actual occurrence of ID. ID is ranked ninth among the 26 modifiable risk factors for death included in the Global Burden of Disease research, and untreated ID leads to IDA [18]. This cross-sectional study intends to determine the actual prevalence of ID and IDA. There is ample research worldwide to estimate the prevalence of anemia and IDA, but only a few studies have used the iron profile as the investigation, which can accurately estimate the actual prevalence of iron insufficiency among the adolescent population. Anemia primarily impacts the vitality and work capacity of women; furthermore, the presence of anemia during pregnancy exerts a detrimental effect on the well-being of the newborn, resulting in intrauterine growth retardation, stillbirth, low birth weight (LBW) infants, and newborn mortality, which are substantial concerns. ID is more common in girls in their later school years and early adolescence. ID causes a variety of non-hematological problems, including stunted growth and development, lowered immunological function in infants, and decreased cognitive function in both infants and adolescents [19]. 

The present study estimated the prevalence rates of anemia as 47.9% (95% CI: 40.2-53.8) among school-going adolescent girls who sought medical care at the tertiary center in Patna, Bihar. Research published by Kulkarni et al., by compiling the reports of NFHS-3, -4, and -5, illustrated the prevalence of anemia as 56.5%, 53.5%, and 59.1%, respectively, which were higher estimates compared to our results. The observed national estimates of the prevalence of anemia deviated from our projected frequency, possibly due to geographical and cultural variation across different states, and also due to the implementation of an institution-based study design and a smaller sample size [8,20-23].

Upon comparing our findings to research conducted in other countries, the occurrence of anemia and ID was significantly lower in developed nations. This disparity can potentially be related to the higher standard of living in those countries and other factors like diet, genetics, etc. Nevertheless, research conducted in impoverished and middle-income countries, such as Pakistan and Saudi Arabia, has demonstrated that the prevalence of anemia is similar to that in India [24-30].

The present study determined the occurrence of ID and IDA in teenage girls (aged 10-19 years) attending school, with a prevalence of 17.8% (95% CI: 11.8%-23.5%) and 15.4% (95% CI: 10.1%-21.7%), respectively. The CNNS, conducted from 2016 to 2018, found that the prevalence of ID in India was 21.5% and in Bihar was 12%, indicating an increase in the estimated prevalence of ID over the period. This is a matter of concern, which could be due to a lack of awareness and non-compliance with iron and folic acid tablet supplementation under the national program [16].

The majority, i.e., 95.1%, of the adolescent girls had ID without the presence of anemia. These girls are likely to develop signs of IDA in the near future, emphasizing the importance of early screening for ID. Likewise, the majority, i.e., 56%, of the adolescent girls with moderate anemia were detected with ID, and only 16% of the girls with mild anemia were detected to have actual ID.

The current results represent a significant association between the severity of anemia and ID status and highlight the importance of investigating other factors contributing to the high prevalence of anemia in Bihar. Kumari et al. conducted a hospital-based study in Patna to estimate the prevalence of IDA among school-going adolescent girls, with a sample size of 200 [31]. It was observed that the mean hemoglobin of the girls aged 10-14 years was 9.8 ± 1.3 g%, and among 15-19 years was 11.3 ± 2.2 g%, which was similar to our estimates. The mean value of serum iron was 58.7 ± 5.6 μg/dL and 72 ± 4.2 μg/dL among the 10-14 years and 15-19 years girls, respectively, and the mean value of serum ferritin was 48.2 ± 2.4 ng/dL and 54.4 ± 3.2 ng/dL. The mean value of TIBC in 10-14 years and 15-19 years of girls was 309 ± 10.8 μg/dL and 277 ± 7.9 μg/dL, respectively. All these estimates were nearly the same as our study estimates [31].

Strengths of the current study include the usage of standard and high-quality procedures for biochemical investigations - such as hemoglobin, serum ferritin, serum iron, percentage saturation of iron, and CRP estimation - thereby accurately assessing the prevalence of absolute ID without inflammation by excluding participants with raised CRP levels. This highlights the critical importance of screening for ID before the clinical presentation of IDA, unlike other studies that used only hemoglobin estimation for determining the prevalence of IDA.

However, the present study has some limitations. The study could have benefited from a community-based approach to better quantify the prevalence of ID and IDA among adolescent girls. It also could not utilize sTfR levels or differentiate between α- and β-thalassemia traits due to the unavailability of these investigations in the study setting. As the study was conducted during the COVID-19 period, it could have potentially affected the selection of study participants. A key limitation is the use of convenience sampling, which may introduce selection bias and limit the generalizability of the findings; employing probability sampling methods could have provided a more representative sample of the adolescent population.

## Conclusions

The findings of this study revealed that almost half of the adolescent females had a high hospital prevalence of anemia, with the majority of these instances classified as mild to moderate in intensity, whereas the hospital prevalence of absolute ID and IDA was low. This highlights the need to determine the underlying cause of anemia and underscores the importance of screening for ID before the actual onset of clinical IDA. The utilization of precise and reliable methods to assess biochemical markers - such as hemoglobin, serum ferritin, serum iron, percentage saturation of iron, and CRP - enhanced the robustness of the analysis, which is a major strength of this study. The hospital prevalence of ID and IDA among adolescent girls highlights the necessity of identifying additional nutritional factors contributing to anemia. This will enable the implementation of focused public health interventions and the prioritization of screening, prevention, and treatment methods to enhance overall health outcomes in adolescent girls.

## Appendices

Questionnaire

Socio-Demographic Profile

CR No: ______________________

● Age of the female student _____________

● Name of the school ___________

● Location of the school

o Rural

o Urban

● Contact number __________________

●Religion

o Hindu

o Muslim

o Christian

o Buddhist

o Jain

o Others

● Caste

o OBC

o SC

o ST

o Others

● Type of family

o Joint (father, mother, children, and others)

o Nuclear (father, mother, and children)

● Number of family members eating from the same kitchen _______________

● Education of the father

o Illiterate

o Literate

o Specify

● Education of the mother

o Illiterate

o Literate

o Specify

● Occupation of the father

o Unemployed

o Farming

o Daily Wages (laborer)

o Industrial/Factory worker

o Govt. Service

o Business/Shopkeeper

o Others

● Occupation of the mother

o Unemployed

o Farming

o Self-Employed

o Daily Wages

o Industrial/Factory worker

o Govt. Service

o Business/Shopkeeper

o Others

● Monthly income of the family ____________________

Personal History

● Have you given blood in the past 3 months?

YES

NO

● Have you transfused blood in the recent 3 months?

YES

NO

● Have you undergone any major surgeries in the past 3 months?

YES

NO

● Is there any history of worm infestation/related diseases (worms visible in the anal area, underwear, or in the toilet, in stools) in recent past 6 months?

YES

NO

● Do you frequently walk barefoot other than in your house?

YES

NO

● Have you attained menarche? If yes, specify the age at which menarche was attained? ________________________________

● What is the length of blood flow during each menses (in days)? ______________

● What do you normally use during menses?

Sanitary pads

Clothes

Menstrual cups

● On average, how many sanitary pads do you use per day? ___________

● On average, how many clothes do you wear per day?

● What is your dietary habit?

Vegetarian

Non-vegetarian

Eggiterian

● Group of food items consumed in the last 24 hours/one day

1. Nuts and seeds (peanuts, almonds, cashew nuts, walnuts, sesame seeds, etc.)

2. Dark green leafy Vegetables (spinach, moringa, fenugreek leaves, etc.)

3. Other vegetables rich in vitamin A (carrot, sweet potatoes, lettuce, etc.)

4. Cereals, roots, plantains and starchy tubers (rice, wheat, barley, ragi, banana, potatoes)

5. Meat, poultry & fish

6. Legumes and pulses (Bean, pea, lentils)

7. Eggs

8. Milk and dairy products

9. Yellow fruits (papaya, mango, etc.)

10. Other fruits (watermelon, guava, etc.)

● Major meal frequency per day

Twice

Three and more

● Post-meal consumption of Tea/Coffee (within 30 min)

Never

Always

Sometimes

● Do you have any of the following symptoms?

1. Easy fatigability (tiredness, loss of strength)

2. Giddiness (tendency to fall)

3. Fainting/syncope (loss of consciousness for a short period of time)

4. Anorexia (loss of appetite)

5. Breathlessness

6. Palpitation

7. Menstrual irregularities (irregular cycle length, bleeding in between cycles)

8. Pica (craving to eat unnatural things like mud, cement, etc.)

9. Do you have any joint pain?

General Examination

● Built and nourishment

o Height of the child (cm)

o Weight of the child (kg)

o Calculated BMI of the child (kg/m2)

● Pallor

o Yes

o No

● Icterus

o Yes

o No

● Cyanosis

o Yes

o No

● Clubbing

o Yes

o No

● Edema

o Yes

o No

● Clinical diagnosis for iron deficiency anaemia

o Glossittis

o Angular stomatitis

o Brittle fingernails

o Flat nails (platynychia)

o Spoon-shaped nails (koilonychia)

o Dysphagia

● Blood sample collected

o Yes

o No

● Blood sample report

o Hemoglobin

o Serum iron

o Serum ferritin

o Total iron binding capacity (TIBC)

o Percentage saturation of iron

o Unsaturated iron binding capacity (UIBC)

o CRP
